# The Breeding Ranges of Central European and Arctic Bird Species Move Poleward

**DOI:** 10.1371/journal.pone.0043648

**Published:** 2012-09-20

**Authors:** Jon E. Brommer, Aleksi Lehikoinen, Jari Valkama

**Affiliations:** 1 Department of Biosciences, University of Helsinki, Helsinki, Finland; 2 ARONIA Coastal Zone Research Team, Novia University of Applied Sciences and Åbo Akademi University, Ekenäs, Finland; 3 Finnish Museum of Natural History, University of Helsinki, Helsinki, Finland; Monash University, Australia

## Abstract

**Background:**

Climatic warming predicts that species move their entire distribution poleward. Poleward movement of the ‘cold’ side of the distribution of species is empirically supported, but evidence of poleward movement at the ‘warm’ distributional side is relatively scarce.

**Methodology/Principal Finding:**

Finland has, as the first country in the world, completed three national atlas surveys of breeding birds, which we here use to calculate the sizes and weighted mean latitudes of the national range of 114 southern and 34 northern bird species during three periods (1974–1979; 1986–1989; 2006–2010), each denoting species presence in approximately 3 800 10×10 km2 squares. We find strong evidence that southern species (breeding predominantly in central Europe) showed a latitudinal shift of 1.1–1.3 km/year poleward during all three pairwise comparisons between these atlases (covering 11, 20.5 and 31.5 years respectively). We find evidence of a latitudinal shift of 0.7–0.8 km/year poleward of northern boreal and Arctic species, but this shift was not found in all study periods and may have been influenced by increased effort put into the more recent surveys. Species showed no significant correlation in changes in range size and weighted mean latitude between the first (11 year) and second (20.5 year) period covered by consecutive atlases, suggesting weak phylogenetic signal and little scope of species characteristics in explaining latitudinal avian range changes.

**Conclusions:**

Extinction-driven avian range changes (at the ‘warm’ side) of a species' distribution occur at approximately half the rate of colonisation-driven range changes (at the ‘cold’ side), and its quantification therefore requires long-term monitoring data, possibly explaining why evidence for such changes is currently rare. A clear latitudinal shift in an assemblage of species may still harbour considerable temporal inconsistency in latitudinal movement on the species level. Understanding this inconsistency is important for predictive modelling of species composition in a changing world.

## Introduction

Understanding factors regulating the distribution of organisms forms the core of ecology. One school of ecologists emphasize the role of abiotic factors, such as temperature, in determining whether an organism can establish a reproducing population at a given site [1], [2],[3]. This Grinellian view is at the heart of species distribution models where present species occurrences are linked to climatic conditions and forward projections are generated on the basis of this information and climate change scenarios (e.g. [4]). According to this view, species extinctions are likely to occur in the coming decades because insurmountable barriers (either geographical or due to habitat fragmentation) hamper species from matching their range to the changing climate [5], [6]. Hence, species are predicted to get “squeezed” to extinction between a rapidly warming climate and a barrier. Another line of research argues that biotic interactions between species are a major driver of species occurrence [7],[8]. Experimental laboratory evidence underlines that when competing *Drosophila* species are considered, changes in distribution are not a simple function of changes in abiotic conditions and an element of unpredictability, caused by species interactions, dominates the system [9]. This view implies that predictions may not be a straightforward resultant of changes in climate and that the consequence of warming for shifts in species' distribution may be specific to the context (e.g. community of species).

Empirical studies on species changing their ranges during recent decades provide only partial evidence for the theories. The majority of studies find indeed that the poleward or high-altitude range margin (“cold” range margin) moves poleward or to higher altitudes, which is consistent with prediction under a climate change scenario [10], [11]. However, the equatorward or low-altitude range margin (i.e. “warm” range margin) typically has not moved [6], [10]. While it should be noted that few studies have jointly considered the warm and cold range margins of the same species assemblage, the empirical evidence – taken together – suggests a range expansion, with few case studies documenting a range retraction. The empirical evidence itself, however, may be biased towards detecting a range expansion. This is because range expansion requires successful colonisation of a new site beyond the current margin by only one individual, but for range retraction a species must become extinct at that site. The biological process of extinction may constitute a relatively long-term process, whereas range expansion (colonisation) is more readily detected [12]. Most empirical demonstrations of range changes consider a relatively short time period. A further issue is that during a certain time period, many confounded environmental changes may occur (e.g. climate warming and eutrophication) and it is thus not always clear which aspect of environmental change is a driver of range changes. One characteristic of climate warming is that it is an ongoing and continuing process. Although this aspect of a continuing process of environmental change is not unique to climate change, we may expect that species' range changes at a given locality should be consistent over time. One approach forward is to use repeated surveys carried out in a similar manner to investigate both short and long-term changes in range and to study the consistency of range changes on the species level. Species-level consistency in range changes over time is required for constructing predictive models or in studying whether range changes covary with certain species characteristics (e.g. dispersal capacity).

In this study, we use the recently completed third atlas of Finnish breeding birds (2006–2010) together with the two previous atlases (1974–79, 1986–89) to study changes in the national range of breeding birds. To our knowledge, this is the first time that three different atlas surveys from the same area have been compared. Each atlas survey is a large citizen science project carried out over four to six years and consists of nationwide mapping of presence/absence of avian species breeding in Finland in approximately 3 800 10×10 km^2^ atlas grid cells. Comparison between the first and second atlas of Finnish breeding birds suggested southern birds, which predominantly breed in central Europe) have shifted their range during this period, but northern (Arctic and boreal zone breeding) birds have not [13]. The third repeat of the atlas survey project allows us to compare changes in the national distribution of the bird assemblage over a maximum period of 3 decades. The signature of climate change in Europe is a particularly rapid increase in temperature during the last 3–4 decades (e.g. [14]). The annual mean temperature in Finland has increased significantly (about 2.1°C) during 1979–2008, and the increase has been particularly rapid in winter (4.3°C) [15]. Hence, we predict that the same assemblage of species has shifted its range further north towards the north pole (hereafter poleward) during the last two decades, possibly at a faster rate. Furthermore, under a climate change scenario, we may expect that species are responding similarly over time. That is, the rate in which species moved poleward during the period covered in the previous analyses is expected to correlate with the rate of change recorded during the more recent survey period.

## Materials and Methods

### Data

Distribution of birds breeding in Finland was obtained from three atlases of Finnish breeding birds; The first atlas reported distribution for 1974–1979 [16], the second atlas presented the distribution for 1986–1989 [17] and the third atlas considered the period 2006–2010 [18]. These atlases use a grid based on 3 859 10×10 km^2^ grid cells. The latitude of grid cells with breeding birds is mapped in these atlases using the Finnish uniform grid which gives the latitude in kilometres north from the equator. For each atlas grid cell, the likelihood that a species bred in the cell is presented in a four-category scale (unlikely, possible, probable and confirmed breeding). The ranking is made by the observer, based on detailed guidelines. For example, direct observation of parents feeding their offspring is considered a confirmed breeding, whereas mere recording of a species denotes a possible breeding. Presence of a species is based on intensive mapping of volunteer ornithologists, in combination with other data sources such as ringing reports (see [17], [18] for more details on the methods). Nevertheless, most of the data came directly from the volunteers using atlas specific forms and e.g. ringing reports contributed a marginal proportion of the total data (e.g. 3% in atlas 3). Each atlas presents the species' national distribution using the maximal amount of information available at the time. As a metric for the amount of information that each atlas contained we calculated the total number of reports contributed by the general public. In the first two atlases, observations were based on a paper form which reported the species observed per atlas grid cell per observer per year. In the third atlas, information was mostly collected electronically using a web-based reporting form which allowed multiple observations per observer per atlas grid cell. To achieve a comparable statistic, we first calculated the number of reports per atlas grid cell per observer per year and then summed this over all years.

We considered the Finnish range size and northern range margins of 114 bird species with a predominantly southern distribution in Finland and 34 species with a northern distribution in Finland. Observations in the category ‘unlikely’ were omitted in our analyses, but all other categories were included. The original selection of species was made by [13] on the basis of the weighted centre grid cell as given in [16]. Basically, these restrictions avoid including species that occur over the entire country and for which a range shift therefore cannot be detected at the scale of Finland. In the comparison between atlas 1 and 2, originally 116 southern species were included [13], but we here exclude the pheasant *Phasianus colchicus* and grey partridge *Perdix perdix*, because the distributions of these species is affected by stocking programmes, which were particularly active in the recent decade.

### Analysis

Finnish range size of each species was estimated as the number of occupied grid cells in each atlas, where a cell was considered occupied if the likelihood of breeding evidence was rated as ‘possible’ or higher. Because there is variation in the number of cells surveyed between atlases (see below), we calculated a statistic of the latitudinal position of a species which takes this variation in the atlas cells surveyed into account. Each atlas covered 115 latitudinal ‘rows’, which, because of the shape of the country, included a variable number of 10×10 km^2^ atlas grid cells. The weighted mean latitude WML of each species *i* was, for each particular atlas survey, calculated as:
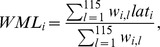
where *lat_l_* is the *l* ^th^ latitude (in Finnish uniform national grid coordinates) and *w_i,l_* the proportion of all surveyed cells at *lat_l_* which were occupied by species *i*. A species' WML weighs its occurrence at a given latitudinal row as the proportion of surveyed cells. Complete absences at a given latitude do not count (*w* = 0) and complete presence in all surveyed cells at a given latitude is given full weight (*w* = 1). This weighting thereby takes into account both the shape of the country (variation in number of surveyed cells across latitude within an atlas) and the variation in number of cells surveyed at each latitude between atlases.

The basic analysis followed the Thomas & Lennon approach [19], where the shift in WML of the assemblage of species is the intercept in a regression of the observed change in WML on the 10-based logarithm of the proportional change in range size. We here used the WML instead of defining the location of the range margin only by the ten most marginal atlas grid cells (as in [19] and [13]), because this latter measure is sensitive to changes in the number of surveyed cells between atlases, which occurred in our case (see Results). In addition, we weighted the linear regression by the log_10_ of species' range size in the most historic atlas of each pairwise comparison. This was done because species with a small range size tended to show large proportional changes which, when not using a weighted regression, have a disproportionally strong influence on the regression coefficients. Log_10_ of species' national range sizes were approximately normally distributed.

To facilitate comparison between atlases, we scaled the change in WML to an annual mean by dividing the observed change with the number of years between the mid-points of each pair of atlases. We thus assume, for simplicity, that changes occur linearly within each study period. Between the first and second atlas, there was an 11 year period (1976/77–1987/88), between the first and the third atlas, there were 31.5 years (1976/77–2008) and between the second and the third atlas there were 20.5 years (1987/88–2008). Between each pair of atlases, we calculated the annual proportional change in Finnish range size *p*. This was scaled to an annual estimate of proportional change in Finnish range size by calculating log_10_(*p* ^(1/*y*)^), where *y* is the number of years between the atlases considered. The log_10_ of the proportional change in Finnish range size between atlases means that no change in Finnish range size has value 0 (proportional change = 1), positive values indicate an expansion and negative values a distributional contraction. The Thomas & Lennon approach thus estimates the extent of the latitudinal change in an assemblage of species which occurs independently from their change in national range size.

## Results

### General survey results

The grid of the atlases of Finnish breeding birds consisted of 3 859 10×10 km atlas cells which were surveyed at least in one of the three atlases. Almost all cells were surveyed during each atlas project, but the number of atlas cells surveyed increased in each consecutive atlas (Table 1). In particular, the survey in the most recent atlas was very complete and presented a clear increase in coverage relative to the previous ones (Table 1). In addition, each consecutive atlas was based on an increasing number of reports, and reporting was especially intensive for the third atlas (Table 1). For southern species (Table S1), the observed range sizes increased during the consecutive atlases (Kruskal-Wallis H = 24.8, P<0.0001) and the weighted mean latitude of the species' assemblage (which takes into account differences in the number of atlas cells surveyed) changed northwards between consecutive atlases (H = 11.6, P = 0.0031). For northern species (Table S2), there was no difference in the mean range sizes (H = 1.74, P = 0.42) and the weighted mean latitudes between the atlases (H = 0.081, P = 0.96;Table 1).

**Table 1:** Information on the survey of the three atlases of Finnish breeding birds and descriptive statistics of the distribution of 114 European bird species which have their northern range margin in southern Finland (southern spp.) and 34 boreal and Arctic bird species with their southernmost distributional margin in northern Finland (northern spp.).

Atlas	Years	N_rep_	Cells	Southern spp. (*n* = 114)		Northern spp. (*n* = 34)	
				Range Sizes	Latitude	Range sizes	Latitude
1	1974–1979	16 036	3726 (96.6%)	1154.6±79.1	6915.2±9.9	549.0±105.9	7480.8±23.4
2	1986–1989	19 951	3745 (97.0%)	1177.0±77.9	6929.8±9.9	585.8±99.8	7472.3±23.8
3	2006–2010	89 227	3848 (99.6%)	1716.0±93.1	6965.2±10.0	783.6±136.7	7469.0±26.3

For each atlas, the total number of reports (N_rep_) and the number of atlas cells surveyed (Cells) are given. A species' national range size was estimated as the number of atlas cells occupied (not corrected for differences in the coverage). ‘Latitude’ is the weighted mean latitude of a species' presence in each atlas, where weighting was based on the proportion of surveyed atlas cells per latitude which were occupied. Latitude is given in Finnish uniform grid coordinates, equivalent to kilometres north of the equator. Means are presented with their standard error.

### Latitudinal change corrected for range change

**Figure 1:**
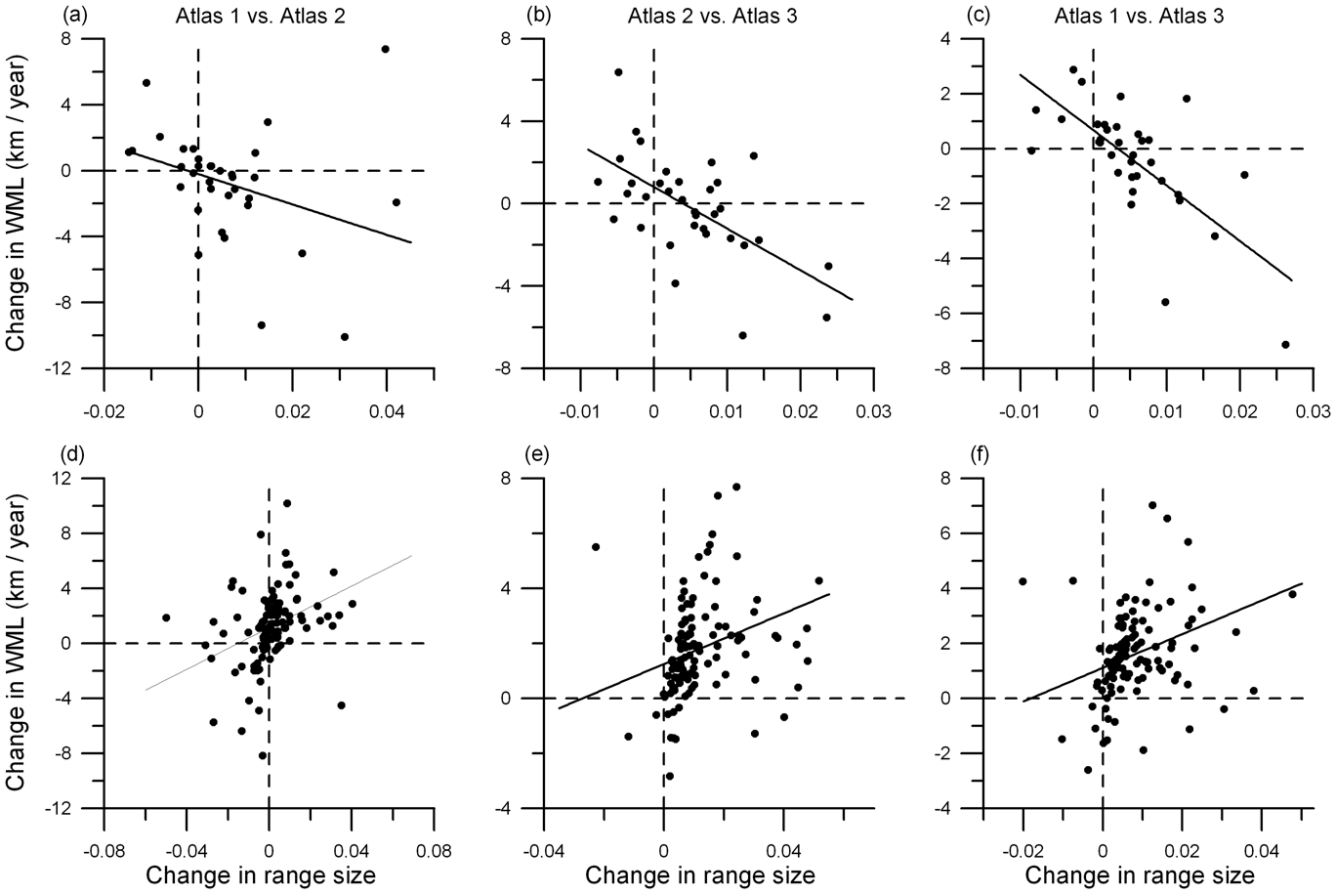
The shift in range margin of 114 European bird species (southern species) and 34 Arctic and northern boreal species (northern species). Plotted is the annual change in weighted mean latitude (in km) against the annual change in range size, as calculated from three atlases of Finnish breeding birds (Table 1). Results are displayed for three pairwise comparisons between the atlases for northern species (upper panels a,b, c) and southern species (lower panels d, e, f). Shift of the assemblage and other regression statistics of the linear regression (solid line) are given in Table 2. Note the differences in the scales across the panels.

In southern species, there was a clear poleward shift in weighted mean latitude between all study periods of approximately 1.1 to 1.3 km/year depending on the time period under consideration (Table 2, Fig. 1d–f). Evidence for a poleward shift of southern species was robust for the correction for multiple testing. The proportional annual change in range size (ΔRange in Table 2) was significantly positively related to the change in mean latitude, as expected for a southern species where increases in the number of occupied cells predominantly occur in northern regions.

**Table 2:** Analyses of the shift in range margin (in km/year) of 114 European birds with their northernmost distributional margin in southern Finland (‘southern’ species) and 34 northern breeding species with their southernmost distributional margin in northern Finland.

Comparison	Assemblage	Property	Estimate (s.e.)	t	P
A1–A2	Southern	**Shift**	**1.14±0.23**	**4.93**	**<0.0001**
(1979–1986)		ΔRange	75.69±20.76	3.65	0.0004
	Northern	Shift	−0.208±0.587	−0.35	0.73
		ΔRange	−92.10±45.05	−2.04	0.049
A2–A3	Southern	**Shift**	**1.25±0.21**	**5.81**	**<0.0001**
(1989–2006)		ΔRange	46.14±14.56	3.17	0.0020
	Northern	Shift	0.810±0.393	2.06	0.048
		ΔRange	−202.10±46.69	−4.63	0.0001
A1–A3	Southern	**Shift**	**1.11±0.18**	**6.03**	**<0.0001**
(1979–2006)		ΔRange	61.27±17.61	3.48	0.0007
	Northern	Shift	0.669±0.306	2.18	0.037
		ΔRange	−201.61±35.63	−5.66	<0.0001

Comparison is between pairwise combinations of the first, second and third atlas (A1, A2, A3, respectively) of Finnish breeding birds (Table 1). Analyses are linear regressions of the annual change in weighted mean latitude between atlases as a function of the logged proportional annual change in range size (ΔRange) weighted by the log_10_ of species' range size in the most historic atlas in each comparison. The ‘shift’ is the intercept of this linear regression and estimates the change in range margin corrected for the change in the other variables. In bold are those shifts which remain significant after Bonferroni-Holm correction for multiple testing [28]. Plots of the analyses are provided in Figure 1.

For northern species, no significant shift was detected in the initial 7 years (atlas 1 vs. atlas 2 in Table 2, Fig. 1d). However, northern species showed a clear tendency for a latitudinal shift of 0.67 km/year northwards in the 31.5 years between atlas 1 and atlas 3 (Table 2, Fig. 1c). Consistent with this finding, northern species also showed a fairly rapid poleward change in mean latitude of 0.81 km/year during the 20.5 years between atlas 2 and atlas 3 (Table 2, Fig. 1b). When correcting for multiple testing of the significance of a shift, there is no overall significance for a latitudinal shift in northern species. The proportional change in range size had a significantly negative effect on the change in mean latitude (Table 2, Fig. 1a–c), which is expected in a northern species. Correcting the observed change in mean latitude for change in range size was clearly important in northern species for proper estimation of the latitudinal shift.

### Consistency of range changes

**Figure 2:**
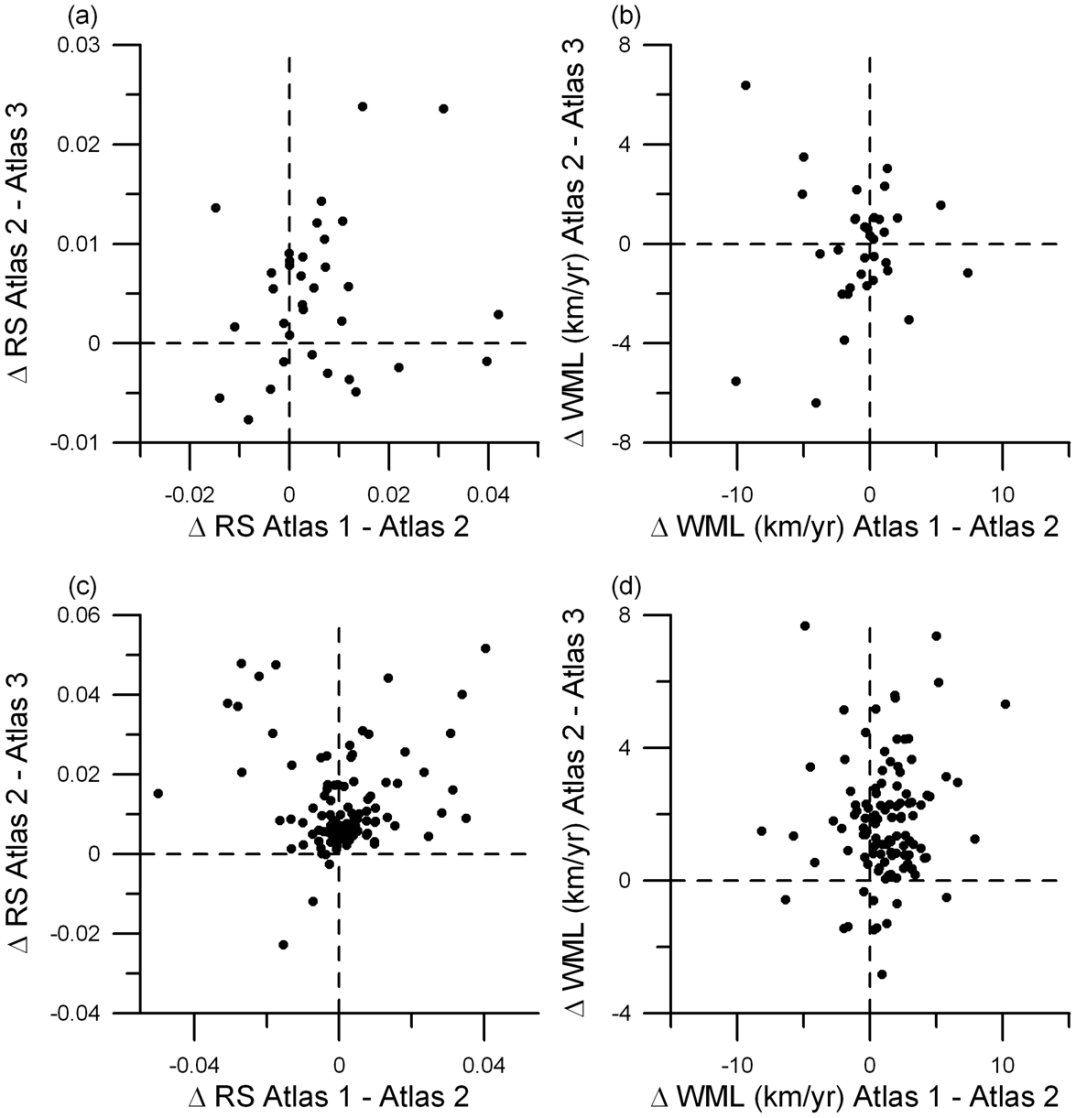
Analysis of the consistency of the change in range size (ΔRS) and change in weighted mean latitude (ΔWML) between the two study periods covered by the three atlases of Finnish breeding birds of 34 northern species (upper panels a and b) and 114 southern species (lower panels c and d). In all panels the change in the latter pair of atlases (atlas 2 vs atlas 3) is plotted against the change in the former pair of atlases (atlas 1 vs. atlas 2). The correlation between these two changes was never statistically significant (see Results).

Under a climate change scenario, we expected that those species which moved their range margin most during the first survey interval (atlas 1 vs. atlas 2) also continued to be moving fastest during the second survey period (atlas 2 vs. atlas 3). There was, however, no significant relationship between these survey periods in either annual change in RS or annual change in WML (Fig. 2). Correlations for the 34 northern species' changes in RS (Fig. 2a) and WML (Fig. 2b) were 0.146 (P = 0.41) and 0.017 (P = 0.92) respectively. Correlations for the 114 southern species' changes in RS (Fig. 2c) and WML (Fig. 2d) were 0.020 (P = 0.83) and 0.122 (P = 0.19) respectively.

## Discussion

We quantified the changes in the distributional patterns of birds breeding in Finland. Importantly, Finland covers a large latitudinal region where many species have their range margin, which thus allows us to study range changes of relatively many species. We here consider 114 southern (central European) bird species which have their ‘cold’ distributional margin in southern Finland and 34 northern (Arctic and boreal) species which have their ‘warm’ distributional margin in northern Finland. Our analyses are based on three national atlases of breeding birds. For southern species, we find large apparent expansions in national range size and clear northwards movements of species in terms of their centre latitudinal coordinate. Also when correcting the latitudinal changes for changes in national range size (i.e. the Thomas & Lennon approach [19] used in several studies of range changes, [20]), we observe a clear significant shift in latitude of southern species. This finding is consistent with what is expected under the hypothesis that climate determines avian species' ranges and has been observed in other regions [20].

We find some evidence that northern species have experienced a similar shift in latitude as southern species have. Poleward movement of the ranges of northern birds is consistent with results from line transect counts in protected areas in Finnish Lapland which suggest that these species are declining in abundance [21],[22]. Our evidence here is mixed. The raw data on latitudinal changes suggests no trend for a southward movement (Table 1). This pattern is, however, affected much by the apparent range expansion of species such that when correcting the change in weighted mean latitude for the change in national range, there is evidence of poleward movement. Statistically, detection of this shift is not robust to corrections for multiple testing. Taken at face value and interpreted as a trend, this finding resembles the pattern found in northerly species in New York state [23]. In our case, the paradoxical situation is that the data on range size suggests that most northern species are undergoing a range expansion (e.g. Fig. 1c where 29 of the 34 species have expanded their observed range 1979–2006). However, when correcting for the change in range size, the mean latitude of the assemblage of northern species moves poleward. This finding indicates that the “shape” of the northern species assemblage's Finnish range is altered such that many sites in the far north are getting colonised. There are a number of interpretations of this finding. First, it must be remembered that one contributing factor in terms of poor statistical significance of the shift of northern Finnish breeding birds is the much lower number of species included in this assemblage compared to southern species (which reflects the fact that species richness in northern boreal and Arctic breeders is lower than in central Europe). Second, one possibly strong factor behind the putative range expansion of northern species is the increased effort put especially into the most recent atlas survey. Variation in effort between atlases partly stems from the fact that the surveying period covered different lengths of time (6, 4 and 5 years for atlas 1, 2 and 3, respectively), but is mostly due to increased interest of potential observers in each consecutive atlas survey projects (Table 1). Atlas cells in the northernmost part of Finland remained relatively frequently unsurveyed during the first atlas (Supplementary Figure 1). The observed range expansion of northern species may thus, partly, be due to a better census. The correction method we here used, which was introduced by Thomas & Lennon [19] in their seminal study on avian range changes, may only partly correct for this variation in effort between atlases. More importantly than changes in coverage, however, is the potential consequences of changes in survey effort in cells that were surveyed in all atlases. The atlases of Finnish breeding birds lack, however, a standard measure of survey effort and we therefore cannot here critically assess to what extent changes in effort (i.e. sampling) and changes in true occupancy are responsible for the pattern we observe. We believe there is some evidence for a latitudinal change also in northern species, but these results should be interpreted with caution and are much less clear than for southern species. Based on the estimates of the latitudinal shift, our findings suggest that whilst we here have the possibility to consider a period that is approximately three times longer (31.5 years between atlas 1 and 3 compared to 11 years between atlas 1 and 2 [13]), we only begin to see evidence of a poleward latitudinal shift of northern species. The rate of the latitudinal shift of the southern part of the distribution of northern boreal and Arctic birds is clearly lower than the rate of the latitudinal shift of the northern part of the distribution of species breeding in central Europe. This result is consistent with the notion that, for whatever reason, extinction-dependent range changes (poleward movement of the ‘warm’ side of the distribution of an assemblage of species) occur on a different time scale than colonisation-dependent range changes (poleward movement of the ‘cold’ side of the distribution).

Our third main finding is that we find low species-level correspondence in range changes between the two study periods covered by the three atlases. Thus, while our results are consistent with a latitudinal shift of the assemblage of species, our findings also imply that this shift harbours considerably temporal heterogeneity at the species level. This low correlation between time periods mirrors the low correlation in range changes of species in Finland and the United Kingdom [20]. There are apparently no characters on the species level that explain range changes in birds, as the rate of range change of the same set of species seems to alter considerably across time (this study) or between regions [20]. Arguably, we lack a clear notion of the strength of the correlation we would expect to find. There is, for example, some resemblance in the latitudinal change of southern species between the two survey periods (Fig. 2d, r = 0.122). This low correlation could reflect what one would expect to find on the basis of large-scale citizen science data, where we cannot properly address changes in data collection between surveys. For example, some species may have been surveyed better in one atlas compared to the other, thereby creating heterogeneity in the observed range changes between study periods. On the other hand, there are also ecological factors which may be responsible for the observed pattern. Competition and predation between the species in an assemblage may promote the range change of a certain species during one period of time, but not in the second period. Furthermore, in a warming world, not only temperature increases, but also other important environmental aspects, such as habitat composition, will change, although at a slower rate. For example, deciduous trees are becoming more common in Finnish forests [24],[25]. These lagged changes are likely to benefit southern forest species more than for example wetland or farmland birds, and may thereby contribute to temporal heterogeneity. Lastly, species may vary in which climatic aspect they respond to most strongly and avian species with certain life histories (e.g. insectivorous migrant) may be more sensitive to climate change than others (e.g. waterfowl) [26],[27], which could also act to generate heterogeneity in their response to climate change over time.

In conclusion, our findings here provide further evidence that species are moving the ‘cold’ side of their range poleward during the last decades. Birds which predominantly breed in central Europe show a strong latitudinal movement towards the Arctic. For northern boreal and Arctic species, we believe we are starting to see a similar poleward latitudinal shift of their breeding range in Finland when investigating changes over almost three decades. We want to be careful in drawing conclusions on the basis of the present findings, because proper estimation and understanding of the shift of northern birds is challenging as there are relatively few species and several confounded factors in our dataset. Nevertheless, if a latitudinal shift in the southern distributional end of northern boreal and Arctic birds indeed occurs, it would be of clear conservation concern, because the northern limit of these species’ breeding ranges is defined by the Ice Sea, a formidable barrier. Taken together, our work suggests latitudinal shifts on the ‘warm’ side of a distribution, which are driven by extinctions of occupied sites, are occurring at a slower rate than what is observed on the ‘cold’ side of a species’ distribution, where changes are driven by colonisation of new sites. Thus, the relatively scarce evidence to date of ‘warm’ range margin shifts compared to ‘cold’ range margin shifts may be due to the relatively short period of monitoring which is usually considered. Long-term monitoring programmes are especially valuable for understanding ongoing distributional changes of northern boreal and Arctic species. Lastly, we need to improve our understanding of the temporal heterogeneity in species' range changes, which we found here. This temporal consistency could be examined in other organisms and in other locations. Clearly, a more detailed study of the potential processes underlying temporal inconsistency in latitudinal and range size changes is required, because understanding such temporal inconsistency has strong implications for our capacity to predict future changes in community structure along latitudinal gradients in a changing world.

## Supporting Information

Figure S1 **Difference in the number of atlas grid cells surveyed for each latitudinal row between atlas 1 and atlas 3 (in blue) and between atlas 2 and atlas 3 (in green).** Atlas 3 is the best surveyed atlas and the red line indicates equal number of cells surveyed as in atlas 3.
(TIF)

Table S1 **The species names (in alphabetical order), range size (RS) and weighted mean latitude (WML) of southern species in the three atlases of Finnish breeding birds (A1, A2, A3, respectively).**
(DOCX)
