# Supplementary figures and images for: The Breeding Ranges of Central European and Arctic Bird Species Move Poleward
Source: PLoS One. 2012 Sep 20;7(9):e43648. doi: 10.1371/journal.pone.0043648 (PMC3447813; doi:10.1371/journal.pone.0043648)

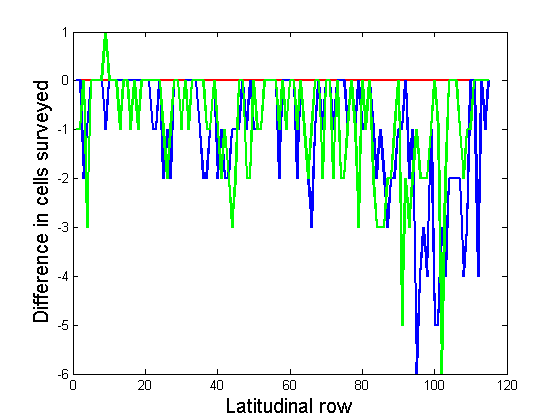

Supplement: Figure S1 — Difference in the number of atlas grid cells surveyed for each latitudinal row between atlas 1 and atlas 3 (in blue) and between atlas 2 and atlas 3 (in green). Atlas 3 is the best surveyed atlas and the red line indicates equal number of cells surveyed as in atlas 3. (TIF) [file pone.0043648.s001.tif]
