# Supplementary material for: The Breeding Ranges of Central European and Arctic Bird Species Move Poleward
Source: PLoS One. 2012 Sep 20;7(9):e43648. doi: 10.1371/journal.pone.0043648 (PMC3447813; doi:10.1371/journal.pone.0043648)
Supplement: Table S1 — The species names (in alphabetical order), range size (RS) and weighted mean latitude (WML) of southern species in the three atlases of Finnish breeding birds (A1, A2, A3, respectively). (DOCX) [file pone.0043648.s002.docx]

**Table S1**. The species names (in alphabetical order), range size (RS) and weighted mean latitude (WML) of southern species in the three atlases of Finnish breeding birds (A1, A2, A3, respectively).

| **Species** | **RS A1** | **RS A2** | **RS A3** | **WML A1** | **WML A2** | **WML A3** |
| --- | --- | --- | --- | --- | --- | --- |
| *Accipiter gentilis* | 1534 | 1743 | 2287 | 7001.2 | 7026.8 | 7066.4 |
| *Accipiter nisus* | 1223 | 1571 | 2288 | 6957 | 7003.8 | 7017.9 |
| *Acrocephalus arundinaceus* | 97 | 96 | 219 | 6721.3 | 6752.5 | 6762.9 |
| *Acrocephalus dumetorum* | 430 | 646 | 1500 | 6842.2 | 6860.5 | 6899.6 |
| *Acrocephalus palustris* | 256 | 525 | 857 | 6769.7 | 6791.4 | 6808.5 |
| *Acrocephalus schoenobaenus* | 1965 | 2023 | 2432 | 7085 | 7088.6 | 7058.1 |
| *Acrocephalus scirpaceus* | 340 | 378 | 496 | 6745.3 | 6754.1 | 6770.4 |
| *Aegithalos caudatus* | 244 | 123 | 1182 | 6895.9 | 6832.6 | 6860.3 |
| *Aegolius funereus* | 1391 | 1782 | 2011 | 6998.9 | 7021 | 7048.6 |
| *Alauda arvensis* | 2362 | 1839 | 2055 | 7027 | 6981.3 | 6992.4 |
| *Anas clypeata* | 655 | 722 | 1016 | 6901.4 | 6923.6 | 6969.5 |
| *Anas platyrhynchos* | 2454 | 2436 | 3173 | 7074.2 | 7077.7 | 7114.7 |
| *Anas querquedula* | 524 | 461 | 495 | 6926.4 | 6938.6 | 6983.5 |
| *Anas strepera* | 22 | 52 | 346 | 6896.5 | 6919.1 | 6905 |
| *Apus apus* | 2432 | 2180 | 2842 | 7078.6 | 7048 | 7084.8 |
| *Ardea cinerea* | 44 | 62 | 500 | 6724.9 | 6760.6 | 6800.8 |
| *Asio otus* | 716 | 823 | 1330 | 6874.4 | 6872.7 | 6882.7 |
| *Aythya ferina* | 661 | 601 | 599 | 6840 | 6855.3 | 6858.5 |
| *Bonasa bonasia* | 2398 | 2078 | 2755 | 7033.2 | 7017.2 | 7072.3 |
| *Botaurus stellaris* | 128 | 82 | 777 | 6760.9 | 6810.7 | 6862.8 |
| *Bubo bubo* | 595 | 1110 | 1368 | 6889.8 | 6908 | 6923.5 |
| *Buteo buteo* | 1892 | 1859 | 2078 | 7004.4 | 7010 | 6980.7 |
| *Caprimulgus europaeus* | 663 | 472 | 715 | 6789.1 | 6770.6 | 6789.2 |
| *Carduelis cannabina* | 777 | 512 | 765 | 6837.2 | 6813.8 | 6846.2 |
| *Carduelis carduelis* | 216 | 106 | 613 | 6768.4 | 6756.4 | 6803.2 |
| *Carduelis chloris* | 1226 | 1526 | 3035 | 6875 | 6987.3 | 7096.5 |
| *Carduelis spinus* | 2466 | 2627 | 3486 | 7046.8 | 7071.7 | 7138.8 |
| *Carpodacus erythrinus* | 1990 | 2131 | 2515 | 6958.8 | 6987.5 | 7003.4 |
| *Certhia familiaris* | 1328 | 1253 | 2370 | 6901.9 | 6898.4 | 6990 |
| *Charadrius dubius* | 840 | 823 | 874 | 6954.3 | 6957 | 6973.8 |
| *Chlidonias niger* | 71 | 20 | 41 | 6870.7 | 6891.2 | 7005.7 |
| *Circus aeruginosus* | 231 | 284 | 1177 | 6812.3 | 6875.3 | 6939.6 |
| *Coccothraustes coccothraustes* | 28 | 61 | 255 | 6866.3 | 6880.3 | 6853.8 |
| *Columba livia* | 433 | 437 | 697 | 6883.2 | 6870.5 | 6911.3 |
| *Columba oenas* | 483 | 453 | 721 | 6772.7 | 6790.6 | 6829.7 |
| *Columba palumbus* | 2241 | 2280 | 2982 | 6973.4 | 7007.8 | 7056.3 |
| *Corvus frugilegus* | 28 | 68 | 104 | 6989 | 6939.4 | 7009.6 |
| *Corvus monedula* | 745 | 722 | 1638 | 6822.8 | 6845.3 | 6932.7 |
| *Crex crex* | 300 | 353 | 1528 | 6808 | 6825 | 6898.5 |
| *Dendrocopos leucotos* | 79 | 45 | 372 | 6820.7 | 6828.6 | 6836.6 |
| *Dendrocopos major* | 2203 | 2465 | 3338 | 7011.1 | 7039.6 | 7127.1 |
| *Dendrocopos minor* | 698 | 643 | 1467 | 7032.8 | 6943 | 6973.4 |
| *Dryocopus martius* | 1597 | 1782 | 2758 | 6969.5 | 7016.9 | 7069.5 |
| *Emberiza citrinella* | 2529 | 2479 | 2924 | 7036.9 | 7044.1 | 7050.1 |
| *Emberiza hortulana* | 1754 | 1459 | 833 | 6969.2 | 6950.8 | 6922.3 |
| *Erithacus rubecula* | 2352 | 2315 | 3176 | 7001.6 | 7013.7 | 7093.6 |
| *Falco subbuteo* | 1126 | 1198 | 2092 | 6932.2 | 6936.9 | 6972 |
| *Falco tinnunculus* | 1489 | 1344 | 2688 | 7033.7 | 7120.7 | 7146.5 |
| *Ficedula parva* | 217 | 238 | 773 | 6810.3 | 6826.5 | 6873.4 |
| *Fringilla coelebs* | 3024 | 2934 | 3341 | 7075.2 | 7071.6 | 7100.2 |
| *Fulica atra* | 756 | 586 | 849 | 6825.8 | 6834.7 | 6881.1 |
| *Gallinula chloropus* | 103 | 52 | 137 | 6774.6 | 6792 | 6809.6 |
| *Garrulus glandarius* | 1823 | 1862 | 2601 | 6951.6 | 6974.1 | 7032.7 |
| *Glaucidium passerinum* | 366 | 394 | 1431 | 6919 | 6913.4 | 6946 |
| *Hippolais icterina* | 491 | 725 | 1014 | 6787.7 | 6809.7 | 6811.3 |
| *Hirundo rustica* | 2826 | 2762 | 3154 | 7049.2 | 7053.3 | 7075 |
| *Jynx torquilla* | 1787 | 1685 | 1943 | 6984.2 | 7018.7 | 7025.9 |
| *Lanius collurio* | 1653 | 1539 | 1999 | 6882.6 | 6887.4 | 6913.6 |
| *Larus argentatus* | 1382 | 1670 | 2474 | 6989.2 | 7014.2 | 7062.2 |
| *Larus canus* | 2130 | 2369 | 3229 | 7080 | 7112.3 | 7128 |
| *Larus fuscus* | 1411 | 1316 | 1711 | 6940.4 | 6935 | 6963.5 |
| *Larus minutus* | 329 | 596 | 1571 | 6992.7 | 7022.7 | 7076.2 |
| *Larus ridibundus* | 2336 | 2298 | 2522 | 7060.3 | 7070.2 | 7012.3 |
| *Locustella fluviatilis* | 120 | 190 | 640 | 6780.6 | 6792.9 | 6838.2 |
| *Locustella naevia* | 430 | 602 | 931 | 6832.8 | 6867.8 | 6875.2 |
| *Loxia curvirostra* | 1858 | 2054 | 2867 | 7067.9 | 7072.2 | 7129.4 |
| *Lullula arborea* | 186 | 85 | 509 | 6763.4 | 6761.8 | 6806.7 |
| *Luscinia luscinia* | 838 | 1020 | 1304 | 6822.8 | 6835 | 6846.5 |
| *Mergus merganser* | 1142 | 1471 | 2174 | 7104.4 | 7121.6 | 7125.4 |
| *Mergus serrator* | 1290 | 1423 | 1776 | 7136.1 | 7131.9 | 7146.4 |
| *Milvus migrans* | 25 | 22 | 69 | 7000.3 | 6946.6 | 7104.2 |
| *Nucifraga caryocatactes* | 70 | 155 | 332 | 6734.4 | 6791.2 | 6913.6 |
| *Numenius arquata* | 2226 | 2203 | 2594 | 7022.6 | 7050.7 | 7058.5 |
| *Oriolus oriolus* | 527 | 467 | 471 | 6816.3 | 6829.2 | 6830.3 |
| *Pandion haliaetus* | 1099 | 1202 | 1944 | 7041.3 | 7070.7 | 7098.5 |
| *Parus ater* | 709 | 734 | 1636 | 6816.3 | 6826.6 | 6894.9 |
| *Parus caeruleus* | 852 | 1180 | 2760 | 6815.3 | 6870.2 | 7021.3 |
| *Parus cristatus* | 1671 | 1601 | 2384 | 6924.4 | 6920.4 | 6958.9 |
| *Parus major* | 2468 | 2568 | 3395 | 7031.6 | 7066.3 | 7141.2 |
| *Parus montanus* | 2516 | 2617 | 3214 | 7071.1 | 7107.3 | 7129.7 |
| *Passer domesticus* | 1997 | 1952 | 2287 | 6985.7 | 7018.4 | 7043.1 |
| *Passer montanus* | 41 | 114 | 1307 | 6780.2 | 6811.9 | 6899.6 |
| *Pernis apivorus* | 1016 | 1137 | 1583 | 6937.2 | 6934.7 | 6965.1 |
| *Phylloscopus collybita* | 2039 | 1883 | 2451 | 6958.2 | 6975.4 | 6999.6 |
| *Phylloscopus sibilatrix* | 1472 | 1883 | 2187 | 6896.5 | 6959.8 | 6949.5 |
| *Phylloscopus trochiloides* | 411 | 295 | 848 | 6836.5 | 6878.6 | 6925.4 |
| *Pica pica* | 2443 | 2531 | 3008 | 7078.1 | 7093.9 | 7119.1 |
| *Picus canus* | 295 | 320 | 1011 | 6727 | 6731.8 | 6837.9 |
| *Podiceps auritus* | 512 | 481 | 767 | 6890 | 6895.3 | 6917.8 |
| *Podiceps cristatus* | 1243 | 1311 | 1458 | 6855.8 | 6893.2 | 6896.8 |
| *Podiceps grisegena* | 453 | 501 | 1183 | 6860 | 6865 | 6918.9 |
| *Porzana porzana* | 386 | 467 | 587 | 6829.1 | 6841.6 | 6885.2 |
| *Prunella modularis* | 1643 | 1823 | 2787 | 7002.9 | 7012.6 | 7072.7 |
| *Pyrrhula pyrrhula* | 2055 | 2128 | 2880 | 7036.3 | 7078.6 | 7098.5 |
| *Rallus aquaticus* | 161 | 101 | 424 | 6754 | 6799 | 6813 |
| *Regulus regulus* | 2041 | 1806 | 2852 | 6990.1 | 6969.1 | 7044 |
| *Saxicola rubetra* | 2491 | 2369 | 2977 | 7069 | 7079.4 | 7101.7 |
| *Scolopax rusticola* | 1728 | 1820 | 2619 | 6909.6 | 6932.6 | 7003.3 |
| *Sterna hirundo* | 1903 | 1906 | 2552 | 6954 | 6981.9 | 7003.5 |
| *Streptopelia decaocto* | 71 | 65 | 209 | 6906.5 | 6895.4 | 6938.5 |
| *Streptopelia turtur* | 130 | 88 | 30 | 6820.2 | 6841 | 6953.9 |
| *Strix aluco* | 698 | 578 | 732 | 6795.5 | 6790.6 | 6783.7 |
| *Strix uralensis* | 732 | 942 | 1624 | 6909.5 | 6911.5 | 6950.7 |
| *Sturnus vulgaris* | 2349 | 1678 | 1785 | 7006.3 | 6936.1 | 6924.2 |
| *Sylvia atricapilla* | 780 | 945 | 1570 | 6801.9 | 6827.7 | 6866.2 |
| *Sylvia borin* | 2223 | 2255 | 2758 | 6972.5 | 6986.5 | 7009.1 |
| *Sylvia communis* | 1669 | 1671 | 2268 | 6877.9 | 6881.9 | 6922.5 |
| *Sylvia curruca* | 2022 | 1906 | 2681 | 6940.7 | 6946.9 | 6985 |
| *Sylvia nisoria* | 75 | 70 | 62 | 6675.9 | 6679 | 6666.6 |
| *Tringa ochropus* | 1614 | 1828 | 2770 | 6986 | 7018.2 | 7066 |
| *Troglodytes troglodytes* | 1039 | 955 | 2064 | 6911.8 | 6908.7 | 6955.9 |
| *Turdus merula* | 1801 | 1498 | 2592 | 6900.4 | 6878.7 | 6984.3 |
| *Turdus viscivorus* | 1342 | 1642 | 3147 | 7032.6 | 7105 | 7165.8 |
| *Vanellus vanellus* | 2336 | 2025 | 2356 | 7042.3 | 7020.6 | 6991 |

**Supplementary Table S2**. The species names (in alphabetical order), their range size (RS) and weighted mean latitude (WML) of northern species in the three atlases of Finnish breeding birds (A1, A2, A3, respectively).

| **Species** | **RS A1** | **RS A2** | **RS A3** | **WML A1** | **WML A2** | **WML A3** |
| --- | --- | --- | --- | --- | --- | --- |
| *Anser fabalis* | 470 | 469 | 719 | 7436.4 | 7410.1 | 7405.1 |
| *Anthus cervinus* | 100 | 70 | 54 | 7688.1 | 7701.3 | 7685.7 |
| *Bombycilla garrulus* | 372 | 539 | 1659 | 7387.9 | 7420.4 | 7358 |
| *Buteo lagopus* | 503 | 656 | 729 | 7573.8 | 7550.7 | 7509.5 |
| *Calcarius lapponicus* | 200 | 200 | 208 | 7660.7 | 7668.4 | 7688.6 |
| *Carduelis flammea* | 1911 | 1858 | 2045 | 7341.8 | 7340.2 | 7352.4 |
| *Charadrius morinellus* | 56 | 76 | 64 | 7653.7 | 7665.5 | 7675.4 |
| *Cinclus cinclus* | 90 | 118 | 211 | 7559.8 | 7541.3 | 7500.1 |
| *Cygnus cygnus* | 467 | 1024 | 3117 | 7388.4 | 7277.3 | 7164 |
| *Emberiza pusilla* | 59 | 171 | 196 | 7500.1 | 7479 | 7399.8 |
| *Falco columbarius* | 639 | 764 | 1253 | 7352.8 | 7350.3 | 7315.8 |
| *Fringilla montifringilla* | 2525 | 2455 | 2248 | 7271.3 | 7285.7 | 7348 |
| *Lagopus lagopus* | 1895 | 1433 | 1549 | 7314.5 | 7373.1 | 7405 |
| *Lagopus mutus* | 60 | 81 | 106 | 7689.9 | 7685.5 | 7674 |
| *Limicola falcinellus* | 224 | 154 | 293 | 7441.5 | 7453.8 | 7501.2 |
| *Loxia leucoptera* | 86 | 235 | 216 | 7302.1 | 7383.3 | 7359.4 |
| *Luscinia svecica* | 484 | 588 | 510 | 7593.5 | 7581 | 7601.1 |
| *Lymnocryptes minimus* | 348 | 317 | 443 | 7454.6 | 7457.1 | 7427.2 |
| *Melanitta nigra* | 143 | 168 | 330 | 7581.4 | 7564.8 | 7528.5 |
| *Numenius phaeopus* | 1075 | 1140 | 1570 | 7411.7 | 7404.4 | 7379.4 |
| *Parus cinctus* | 393 | 472 | 678 | 7543.5 | 7539.2 | 7553.2 |
| *Perisoreus infaustus* | 749 | 801 | 1207 | 7400.7 | 7388.6 | 7409.7 |
| *Phalaropus lobatus* | 366 | 332 | 267 | 7547.3 | 7536.4 | 7581.2 |
| *Philomachus pugnax* | 1026 | 833 | 580 | 7350.9 | 7373.7 | 7395.2 |
| *Phylloscopus borealis* | 87 | 122 | 97 | 7458.7 | 7355.6 | 7486.4 |
| *Pinicola enucleator* | 278 | 256 | 332 | 7540.8 | 7555.4 | 7533.6 |
| *Plectrophenax nivalis* | 70 | 75 | 88 | 7663.8 | 7667 | 7688.8 |
| *Pluvialis apricaria* | 1076 | 1219 | 1586 | 7397.6 | 7356.3 | 7348 |
| *Stercorarius longicaudus* | 139 | 156 | 148 | 7697 | 7697 | 7703.7 |
| *Strix nebulosa* | 201 | 231 | 409 | 7273 | 7228.1 | 7097 |
| *Surnia ulula* | 397 | 693 | 618 | 7386.5 | 7331.3 | 7403 |
| *Tringa erythropus* | 522 | 557 | 669 | 7497.5 | 7500.6 | 7504.3 |
| *Tringa nebularia* | 1635 | 1634 | 2415 | 7283.5 | 7286.7 | 7276.4 |
| *Turdus torquatus* | 20 | 20 | 29 | 7703.8 | 7647.6 | 7688.8 |
